# Prochlorperazine dimaleate elicits antiproliferative activity of gastric cancer cells via inhibiting the PI3K/AKT/mTOR signaling pathway

**DOI:** 10.1016/j.gendis.2026.102032

**Published:** 2026-01-07

**Authors:** Yunhao Ma, Zhenzhen Si, Zhongkun Zhou, Yuanchun Zhao, Yanan Tian, Xuanru Zhang, Huanxiang Liu, Hongmei Zhu, Yingqian Liu, Peng Chen

**Affiliations:** a School of Pharmacy, Lanzhou University, Lanzhou, Gansu 730000, China; b State Key Laboratory of Applied Organic Chemistry, Lanzhou University, Lanzhou, Gansu 730000, China; c Faculty of Applied Sciences, Macao Polytechnic University, Macao SAR 999078, China

Gastric cancer is a common malignant tumor worldwide.^1^ Although chemotherapy drugs are effective against some solid tumors, their clinical application is limited due to the toxic side effects on normal tissues and drug resistance.^2^ The development of traditional drugs is expensive and time-consuming. Therefore, the concept of drug repurposing has been proposed and drug repurposing is gaining widespread attention as a strategy for drug development.^3^^,^^4^ One promising strategy is to repurpose approved clinical drugs for novel anti-gastric cancer applications, which has obvious advantages, including prior knowledge of drug safety and dosage, making the development of anti-cancer drugs faster and cheaper^5^ Here, we report an efficient strategy for repurposing previously approved drugs with novel indication. Prochlorperazine dimaleate (PD) (Fig. 1A) is a phenothiazine antipsychotic that also exhibits antiemetic and anti-allergic effects. PD showed potent antitumor effects in gastric cancer both *in vitro* and *in vivo*, and its mechanism and targets were further explored. These results suggest that PD may be a promising candidate for the development of anti-gastric cancer drugs. PD inhibited the growth of gastric cancer AGS and HGC27 cells in a concentration- and time-dependent manner. The IC_50_ values of PD against AGS (Fig. S1A) and HGC27 (Fig. S1D) cells were 6.00 μM, 4.61 μM at 48 h respectively. PD also showed cytotoxic effects in other cancer cell lines (Fig. 1B). Specifically, the IC_50_ values of PD in pancreatic cancer PANC-1 cells, lung cancer A549 cells, breast cancer MDA-MB-231 cells, liver cancer SMMC7721 cells, melanoma B16F10 cells, cervical cancer HeLa cells, colorectal cancer HCT116 cells, gastric cancer HGC27, MKN45, MGC803, SGC7901 cells and gastric mucosal GES-1 cells at 48 h were 24.10, 15.63, 20.45, 15.19, 3.90, 11.70, 6.13, 4.61, 6.55, 8.38, 22.74, 29.00 μM, respectively (Fig. 1C). In summary, PD showed potent cytotoxic effects in gastric cancer cells and exhibited low cytotoxicity toward normal gastric mucosa cells.

**Figure 1:**
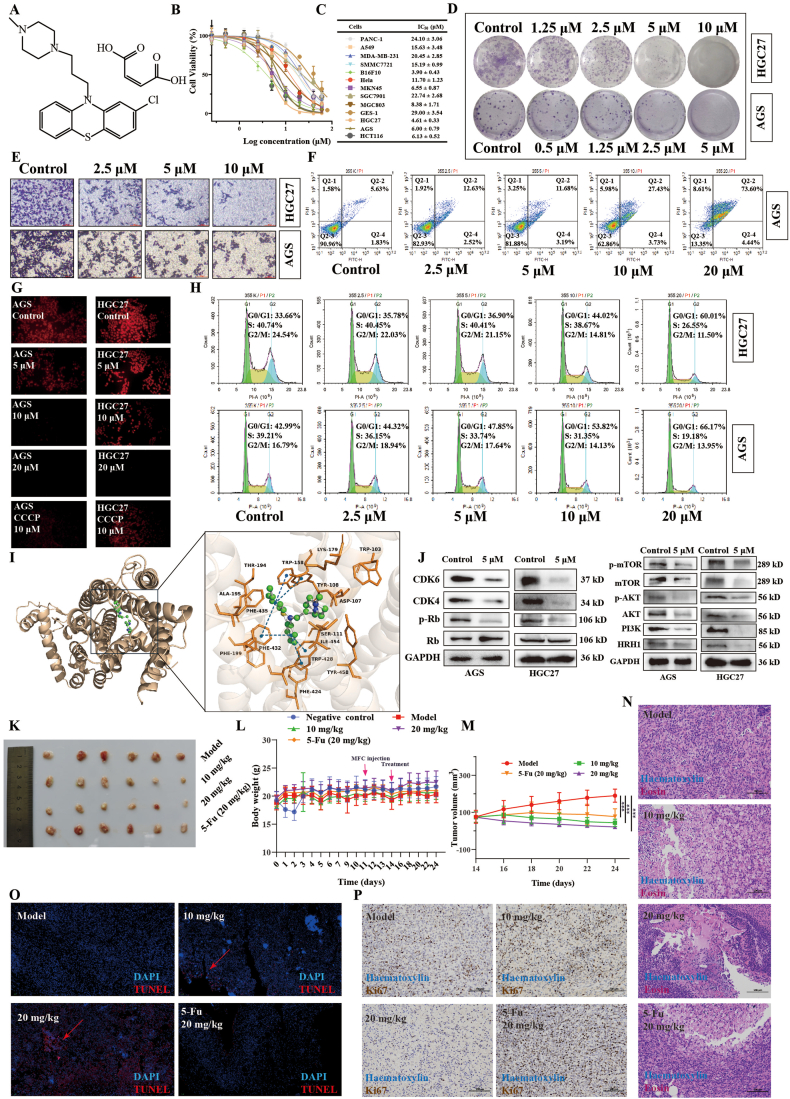
The biological effects and mechanisms of prochlorperazine dimaleate (PD) in gastric cancer therapy by the PI3K/AKT/mTOR signaling pathway. **(A)** Chemical structure of PD. **(B)** The cell viability after treatment with different concentrations of PD for 48 h. **(C)** IC_50_ values of PD against PANC-1, A549, MDA-MB-231, SMMC7721, B16F10, HeLa, MKN45, SGC7901, MGC803, GES-1, HGC27, AGS and HCT116 cells at 48 h. **(D)** The colony formation assay of AGS and HGC27 cells treated with different concentrations of PD for 8–10 days. **(E)** The migration capacity of gastric cancer AGS and HGC27 was inhibited after the treatment of PD for 48 h. **(F)** Cell apoptosis analysis in AGS cells treated with different concentration of PD for 48 h. **(G)** The mitochondrial membrane potential of AGS and HGC27 cells was reduced after treatment with PD for 48 h. **(H)** PD arrested AGS and HGC27 cells at G0/G1 phase after treatment for 24 h. **(I)** Detailed interaction between PD and the HRH1 protein. **(J)** PD inhibited the expression of proteins associated with cells cycle and the PI3K/AKT/mTOR cell signaling pathway in AGS and HGC27 cells. **(K)** The tumor volumes of BALB/c mice after the treatment of PD or 5-Fluorouracil (5-Fu). **(L)** The body weight of BALB/c mice before and after administration of PD or 5-Fu. **(M)** The changes of tumor volumes of BALB/c mice after the treatment of PD or 5-Fu. **(N)** The hematoxylin and eosin (HE) staining of tumor tissues in different groups after sacrifice. **(O)** TUNEL staining for apoptosis analysis of tumor tissues in different groups after sacrifice. **(P)** The proportion of Ki67-positive cells in immunohistochemistry experiments among different groups. Values are shown as the means ± standard deviation, *n* = 3. ∗*p* < 0.05, ∗∗*p* < 0.01, ∗∗∗*p* < 0.001 compared to negative control group.

In subsequent experiments, PD was found to inhibit colony formation in gastric cancer AGS and HGC27 cells (Fig. 1D). After treatment with PD at concentrations of 1.25 μM, 2.5 μM, 5 μM, and 10 μM (for AGS cells) or 0.5 μM, 1.25 μM, 2.5 μM, and 5 μM (for HGC27), the number of colonies decreased from 192 to 2 in AGS cells and from 106 to 4 in HGC27 cells, respectively (Fig. S1B and S1E). We also evaluated the migration capacity of gastric cancer AGS and HGC27 cells after 48 h of treatment with PD (2.5 μM, 5 μM, 10 μM) (Fig. 1E). After the treatment, the number of migrating HGC27 cells decreased from 70 to 14, and that of AGS cells decreased from 126 to 56 (Fig. S1C and S1F).

Regulating the cell cycle and inducing cell apoptosis are common mechanisms through which anti-tumor drugs exert their effects. After 48 h of treatment with PD, AGS and HGC27 cells exhibited nuclear contraction and obvious apoptotic characteristics compared to the 5-Fluorouracil (5-Fu) group (Fig. S2A). PD induced apoptosis in AGS (Fig. 1F) and HGC27 (Fig. S2F) cells in a concentration-dependent manner. The apoptotic rates of AGS and HGC27 cells were increased from 6.90% to 71.46% and from 4.90% to 78.34%, respectively (Fig. S2D and S2E). The results of TMRE staining assay showed that red fluorescence of the AGS and HGC27 cells treated with PD gradually decreased, and the mitochondrial membrane potential of the cells decreased compared with the control and 5-Fu groups (Fig. 1G). After the treatment of PD, AGS and HGC27 cells were arrested at G0/G1 phase (Fig. 1H). The ratio of cells in G0/G1 phase were increased from 37.02% to 59.67% in HGC27 cells and from 44.82% to 64.16% in AGS cells, respectively (Fig. S2B and S2C).

To identify the potential targets of PD, small molecule PD were docked with a series of proteins involved in the cell cycle, apoptosis and signaling pathway (Table S1). PD showed the strongest interaction with HRH1 (PDB: 3rze) and the docking score was −9.74. PD effectively competed with histamine binding and inhibited its functional activity by binding to the normal binding site of HRH1 (Fig. 1I). The binding of PD to the receptor involves multiple forces including hydrophobic interaction, π-π interaction, polarity interaction and ionic bond interaction (Fig. S3B). Kaplan–Meier survival analysis indicated that high expression of HRH1 was associated with poor prognosis of gastric cancer patients (Fig. S3A). Data from TCGA and GTEx databases showed increased levels of HRH1 mRNA in gastric cancer tissues (Fig. S3C). Next, the expression of HRH1 and the proteins related cells cycle and PI3K/AKT/mTOR cell signaling pathways were evaluated (Fig. 1J). The expression levels of cell cycle-related proteins CDK6, CDK4, p-Rb in AGS and HGC27 cells decreased by 0.43, 0.53, 0.38 (AGS) and 0.41, 0.48, 0.65 (HGC27) (Fig. S3D and S3E). The decreased expression of these proteins further confirmed our results that PD can block AGS and HGC27 cell cycles at the G0/G1 phase. The HRH1 protein levels in AGS and HGC27 cells were decreased by 0.49 and 0.71 respectively after the treatment of PD (5 μM) for 48 h (Fig. S3F and S3G). Furthermore, expression of PI3K, AKT, p-AKT, mTOR and p-mTOR in AGS and HGC27 cells were decreased by 0.64, 0.67, 0.66, 0.38, 0.54 and 0.32, 0.57, 0.69, 0.43, 0.73, 0.71 respectively (Fig. S3F and S3G). These results suggest that PD inhibits the proliferation of AGS and HGC27 cells by regulating the PI3K/AKT/mTOR cell signaling pathway.

The efficacy of PD in suppressing the growth of xenograft tumors was evaluated in a relevant tumor model (Fig. S4A). Mice in the treatment group received intravenous injections of 10 mg/kg or 20 mg/kg PD, or 20 mg/kg 5-Fu, every other day. Our results showed that the tumor volume and weight were reduced in the treated group, but no significant change in the body weight was observed compared with the control group (Fig. 1K–M; Fig. S4B). No significant differences were found in the weights of the heart and kidney among different groups (Fig. S4C–S4G). Moreover, hematoxylin and eosin (HE) staining analysis of the main organs (heart, liver, spleen, lung, and kidney) also revealed no obvious pathological changes in the main organs of any treatment groups (Fig. S4H). HE staining of tumor tissues showed that the interstitial space between the nuclei increased after the treatment of PD (Fig. 1N). The proportion of Ki67-positive cells was higher in the model group, but the proportion of Ki67-positive cells significantly decreased after the treatment of PD, suggesting that PD can inhibit gastric cancer cell proliferation (Fig. 1P). The results of the TUNEL assay showed that the proportion of cells with red fluorescence (apoptotic cells) increased after the treatment with PD, indicating that PD promoted the apoptosis of tumor cells and thereby inhibited the proliferation of gastric cancer cells *in vivo* (Fig. 1O).

In conclusion, this study demonstrates that PD, an antipsychotic agent, can inhibit the proliferation of gastric cancer cells both *in vitro* and *in vivo*. Mechanistically, PD exerted cytotoxic effects in gastric cancer cells by targeting HRH1 and medicating the PI3K/AKT/mTOR signaling pathway. Overall, our findings supported PD as a promising candidate for the treatment of gastric cancer.

## CRediT authorship contribution statement

**Yunhao Ma:** Writing – review & editing, Writing – original draft, Investigation, Formal analysis, Data curation. **Zhenzhen Si:** Methodology, Investigation, Data curation. **Zhongkun Zhou:** Visualization, Validation, Methodology. **Yuanchun Zhao:** Software, Methodology, Investigation. **Yanan Tian:** Investigation, Data curation. **Xuanru Zhang:** Validation, Methodology. **Huanxiang Liu:** Methodology, Formal analysis. **Hongmei Zhu:** Validation, Methodology. **Yingqian Liu:** Validation, Methodology. **Peng Chen:** Writing – review & editing, Supervision, Project administration.

## Ethics declaration

The animal experiments were carried out following the procedures of the Reporting of In Vivo Experiment (ARRIVE) guidelines and using a protocol approved by the Institutional Animal Ethics Committee of the School of Pharmacy, Lanzhou University (Approval date: 2025-06-06).

## Funding

This work was supported by Gansu Provincial Science and Technology Major Project, China (No. 24ZDFA001), the Lanzhou Municipal Science and Technology Program, China (No. 2024-4-2, 2025-2-100, 2025-2-103) and the College Students' Innovation and Entrepreneurship Program of Lanzhou University, China (No. 20250260006, 20250260016, 20250260020).

## Conflict of interests

The authors declare that there is no conflict of interests.
