# Supplementary material for: Prochlorperazine dimaleate elicits antiproliferative activity of gastric cancer cells via inhibiting the PI3K/AKT/mTOR signaling pathway
Source: Genes Dis. 2026 Jan 7;13(6):102032. doi: 10.1016/j.gendis.2026.102032 (PMC13355024; doi:10.1016/j.gendis.2026.102032)
Supplement: Multimedia component 1 [file mmc1.docx]

**Supplementary Information for**

**Repurposed** **perphenazine inhibited the growth of gastric cancer cells by regulating PI3K/AKT signaling pathway**

Yunhao Ma^1, 2, †^, Zhenzhen Si^1, 2, †^, Zhongkun Zhou^1, 2^, Yuanchun Zhao^1, 2^, Yanan Tian^3^, Xuanru Zhang^1, 2^, Huanxiang Liu^3^, Hongmei Zhu^1^, Yingqian Liu^1, *^, Peng Chen^1, 2, *^

^1^ School of Pharmacy, Lanzhou University, No. 199 Donggang West Road, Lanzhou, 730000, PR China

^2^ State Key Laboratory of Applied Organic Chemistry, Lanzhou University, Lanzhou 730000, PR China.

^3^ Faculty of Applied Sciences, Macao Polytechnic University, Macao, SAR, China

* Corresponding author:

Correspondence and requests for materials should be addressed to Peng Chen (email: chenpeng@lzu.edu.cn) or Yingqian Liu (email: yqliu@lzu.edu.cn). Tel. & fax: +86 931 8915686.

**This PDF includes:**

Material and methods

Table S1

Figure S1-S4

**Material and methods**

**Reagents and antibodies**

Prochlorperazine Dimaleate was purchased from Bide Pharmatech Ltd. (Cat# BD376942), GAPDH (1:2000), CDK4 (1:2000), CDK6 (1:2000), Rb (1:2000), p-Rb (1:200) were bought Sangon Biotech Co., Ltd. (Cat # D110016, Cat# D120396, Cat# D120398, Cat# D151675, Cat# D155057). PI3K (1:2000) was obtained by Affinity Biosciences (Cat# AF6242). AKT (1:2000), p-AKT (1:2000), mTOR (1:2000), p-mTOR (1:2000) were bought from Immunoway (Cat# YT0185, Cat# YP0006, Cat# YT2913, Cat# YP0176). RPMI 1640, DMEM medium were purchased from Biological Industries (Cat# C3010 and Cat# C3130), 0.25% Trypsin solution was obtained from KeyGEN BioTECH Corp. Ltd (Cat# KGL2102). Streptomycin/penicillin solution were purchased from New Cell & Molecular Biotech Co., Ltd (Cat# C100C5); Annexin V-Alexa Fluor 488/PI Kit, DNA Content Detection Kit, DAPI Dye were purchased from Solarbio (Cat# CA1040, Cat# CA1510, Cat# C0065).

**Cell** **culture**

Human gastric cancer AGS cells, colorectal cancer HCT116 cells and pancreatic cancer PANC-1 cells were derived from the American Type Culture Collection (ATCC), AGS (LOT:70012225), HCT116 (LOT:70019042), PANC-1 (LOT:70018880). Human lung cancer A549 cells, human breast cancer MDA-MB-231 cells, human liver cancer SMMC-7721 cells, Melanoma B16F10 cells, human cervical cancer Hela cells and human gastric cancer MKN45, SGC7901, MGC803, HGC27 cells and human gastric mucosa GES-1 cells were derived from the genetic resource library of our laboratory. A549, SMMC-7721, B16F10, Hela, MKN45, HGC27, AGS and HCT116 cells were cultured in RPMI and PANC-1, MDA-MB-231, SGC7901, MGC803 and GES-1 cells were cultured in DMEM medium containing 10% serum and 1% penicillin and streptomycin. These cells were cultured at 37 °C in humidified atmosphere of 5% CO_2_ in air.

**Cell viability assay**

Different cancer cells were seeded in 96-well plates at 8-10 × 10^3^ cells/well for 24 h, and then treated them with PD for 24 h, 48 h, 72 h. The antiproliferative activities of PD against different cancer and normal cell lines were monitored using MTT methods.10 μL MTT solution (5 mg/mL) was added for further incubation for 4 h. The absorbance values were measured using a microplate reader at 490 nm, and the inhibition rate of the PD was calculated to evaluate the toxicity of PD. The mean value of triplicate experiments for each dose was used to represent the cell viability.

**Colony formation**

The human gastric cancer cells (AGS or HGC27) cells were seeded in 24-well cell culture plates and treated with PD at the indicated doses (0.5 μM, 1.25 μM, 2.5 μM, 5 μM, 10 μM).

The cells were treated with the PD for about 8-10 days. Cells were washed with PBS twice and fixed with 4% paraformaldehyde solution for 40 min followed by staining with 1% crystal violet dye at room temperature for 20 min. The 24-well plates were then rinsed with distilled water and dried prior to scanning.

**Cells migration assay**

600 μL cells culture medium containing 20% FBS was added in 24-well plate. Human gastric cancer AGS and HGC27 cells suspension with 1% FBS and PD (2.5 μM, 5 μM, 10 μM) were seeded in the transwell chamber at a density of 3 × 10^4^ cells per well. The AGS and HGC27 cells were treated with PD for 48 h and fixed with 4% paraformaldehyde solution. After the cells were stained with 1% crystal violet dye, the upper part of transwell chamber was dried with cotton swabs and scanned under a microscope. The results were statistically analyzed by image J. The mean value of triplicate experiments for each dose was showed.

**Cell cycle assay**

The DNA content analysis was determined by staining DNA with propidium iodide (PI). The operation procedure was according to the protocol of DNA content quantitation assay (Cell cycle) kit (Solarbio, Cat# CA1510). After incubation with PD (2.5 μM, 5 μM, 10 μM and 20 μM) for 24 h, AGS and HGC27 cells were harvested and then washed in PBS twice and fixed in 70% ethanol. The ethanol was washed away and cells were treated in 100 μL Rnase at 37 °C for 30 min. Finally, cells were incubated with PI (400 μL). Then the cell cycle distribution and data processing were detected by flow cytometry (Agilent NovoCyte Penteon, Singapore). The fluorescence profiles represent the DNA content of the PI stained cells.

**Cell apoptosis**

The apoptotic cells were determined by an Annexin V Alexa Fluor488/PI double staining apoptosis kit (Solarbio, Cat# CA1040). Succinctly, AGS and HGC27 cells were treated with PD (2.5 μM, 5 μM, 10 μM and 20 μM) for 48 h, then digested and collected for the following staining. The binding buffer was used to wash and resuspend the cell suspension. 5 μL Annexin V/Alexa Fluor 488 and PI staining solution were then added the cells and incubated for 10 min at room temperature in the dark. The apoptosis cells were acquisition using flow cytometry (Agilent NovoCyte Penteon, Singapore).

**DAPI staining and mitochondrial membrane potential assay**

AGS and HGC27 cells were seeded in the 24-well plate for 24 h and were treated with PD (5 μM, 10 μM and 20 μM) or 5-Fu (20 μM) for 48 h. Then the cells were fixed with 4% paraformaldehyde solution, and stained with DAPI staining solution (Coolaber, Cat# SL7100) for 5-10 min at room temperature in the dark. The nuclear morphology was captured by fluorescence microscope (Olympus BX53+DP74, Japan). The mitochondrial membrane potential was assessed by tetramethylrhodamine ethyl ester (TMRE, Beyotime Biotechnology, C2001S) staining. AGS and HGC27 cells were treated with different concentrations (5 μM, 10 μM and 20 μM) of PD for 48 h and then treated with positive drug Carbonyl cyanide 3-chlorophenylhydrazone (CCCP, 10 μM) for 20 min. Then the AGS and HGC27 cells were stained with TMRE staining solution (1×) at 37 °C for 30 min. Images were captured using a fluorescence microscope (Olympus BX53+DP74, Japan) to visualize and evaluate mitochondrial membrane potential.

**Molecular docking analysis**

The crystal structures of Histamine H1 Receptor protein (PDB: 3rze) bound to molecules in docking were obtained from the PDB database. Molecular docking was performed by using Schrödinger 10.1 software (Schrödinger, USA). First, we removed the complex protein structure of water of crystal, side chain and hydrogen atom, and processed protein with minimal energy. Then, the center binding to the protein was used as the docking site of PD and the proteins for analysis.

**Bioinformatics analysis**

The Gene Expression Profiling Interactive Analysis (GEPIA2, http://gepia2.cancer-pku.cn/) database was used to explore the differences in RNA-seq expression data of Histamine H1 Receptor between tumor and normal tissues, retrieved from TCGA and GTEx databases. In addition, to assess the prognostic value of Histamine H1 Receptor, Kaplan–Meier survival analysis was employed using the Kaplan–Meier Plotter online tool (https://kmplot.com/analysis/) to identify differences in overall survival between the high-and low-expression groups.

**Western blotting assay**

The AGS and HGC27 cells were harvested and lysed in RIPA buffer (Solarbio, Cat# R0010) with 1% phenylmethanesulfonyl fluoride (PMSF) and phosphatase inhibitor buffer on ice for 30 min. The protein concentration was detected using BCA protein assay kit (Coolaber, Cat# SK1070). Approximately 25 μg of protein per sample for gastric cancer cells were separated by 12% SDS-PAGE gel and transferred to PVDF membranes (Biosharp, MerckMillipore ISEQ00010). The membrane was then blocked with 5% non-fat milk for 2 h at room temperature. The targeted proteins were incubated overnight at 4 °C with the following specific primary antibodies: CDK4, CDK6, Rb, p-Rb, PI3K, AKT, p-AKT, mTOR, p-mTOR, GAPDH. Then, the membranes were washed and exposed to HRP-conjugated secondary antibody for 2 h at room temperature. Finally, the membranes were detected with an enhanced chemiluminescence detection kit (New Cell & Molecular Biotech Co., Ltd, Cat# P10200). The total density of the protein bands was detected using the Tanon imaging system (Tanon Science & Technology Co., Ltd). Image J was used for gray value analysis. All experiments were repeated for three times and averaged for analysis.

**Tumor xenograft experiments**

The animal experiments were carried out following the procedures approved by Reporting of In Vivo Experiment (ARRIVE) guidelines the protocol approved by the Institutional Animal Ethics Committee of School of Pharmacy, Lanzhou University (Approval date: 2025-06-06). Female BALB/c mice aged 6-8 weeks were maintained Lanzhou animal research institute, Gansu province, China (SCXK (Gan)-2020-0002). BALB/c mice were subcutaneously injected with MFC (3, 000, 000 cells) were resuspended in PBS were mixed with Matrigel in equal volume. Once the tumor volume reached 80-100 mm^3^, the mice were divided into different groups and treated with the PD or 5-Fu every other day. After transplantation, the tumor size was measured every other day with calipers, and the tumor volume was estimated by the following formula: tumor volume (mm^3^) = 0.5 × (L × W^2^). The treatment was continued about two weeks before sacrifice, the tumors and major organs of mice were analysis.

**Quantification and statistical analysis**

The data were analyzed using SPSS22.0 statistical software, GraphPad PriM 8.0 and AI2020. All values were showed as the mean ± SD. The statistical differences between two groups were determined by the student’s *t*-test. Values of *p* ≤ 0.05 were used as the criterion for statistical significance.

**Table S1. The molecular docking scoring of PD and protein**

| Target | PDB ID | Resolution(Å) | Organisms | Docking score |
| --- | --- | --- | --- | --- |
| Histamine H1 Receptor | 3rze | 3.1 | *Home sapiens* | -9.74586 |
| AKT | 6hhf | 2.9 | *Home sapiens* | -8.19991 |
| CyclinE1 | 5l2w | 2.8 | *Home sapiens* | -7.63762 |
| Dopamine Receptor D2 | 6cm4 | 2.87 | *Home sapiens* | -7.25078 |
| mTOR | 4jt6 | 3.6 | *Home sapiens* | -7.1714 |
| CDK2 | 5nev | 2.97 | *Home sapiens* | -7.00488 |
| MAPK | 2g01 | 3.5 | *Home sapiens* | -6.84306 |
| CDK6 | 4ez5 | 2.7 | *Home sapiens* | -6.82371 |
| JNK | 4y5h | 2.055 | *Home sapiens* | -6.75932 |
| MEK | 5eym | 2.7 | *Home sapiens* | -6.75188 |
| PARP | 6bhv | 2.3 | *Home sapiens* | -6.40426 |
| CDK1 | 6gu2 | 2 | *Home sapiens* | -6.35226 |
| Alpha-2A adrenergic receptors | 6kux | 2.7 | *Home sapiens* | -6.3511 |
| CDK7 | 8p4z | 2.75 | *Home sapiens* | -6.22056 |
| PI3K | 6gvf | 2.5 | *Home sapiens* | -6.15952 |
| Raf | 5fd2 | 2.89 | *Home sapiens* | -5.89663 |
| Alpha-1 adrenergic receptors | 7ymj | 3.35 | *Home sapiens* | -5.82205 |
| Bcl-2 | 6qgg | 1.5 | *Home sapiens* | -5.68913 |
| caspase-3 | 3dej | 2.6 | *Home sapiens* | -5.67192 |
| JAK | 5tq6 | 2.06 | *Home sapiens* | -5.55233 |
| Bcl-xl | 7lh7 | 1.409 | *Home sapiens* | -5.38229 |
| CyclinD1 | 5vzu | 2.7 | *Home sapiens* | -5.30373 |
| Erk5 | 6hkn | 2.33 | *Home sapiens* | -4.84207 |
| caspase-9 | 1nw9 | 2.4 | *Home sapiens* | -4.40843 |
| STAT3 | 6nuq | 3.15 | *Home sapiens* | -4.40272 |
| CDK4 | 3g33 | 3 | *Home sapiens* | -4.19807 |
| NF-kB | 1a3q | 2.1 | *Home sapiens* | -4.02172 |
| CyclinA | 6gue | 1.99 | *Home sapiens* | -3.65323 |
| GSK-3β | 1pyx | 2.4 | *Home sapiens* | -3.2648 |
| β-catenin | 7afw | 1.81 | *Home sapiens* | -2.88181 |
| E-cadherin | 4zte | 2.13 | *Home sapiens* | -2.87189 |
| Wnt | 6gbi | 1.25 | *Home sapiens* | -2.72177 |
| Ras | 7jhp | 2.766 | *Home sapiens* | -2.06565 |


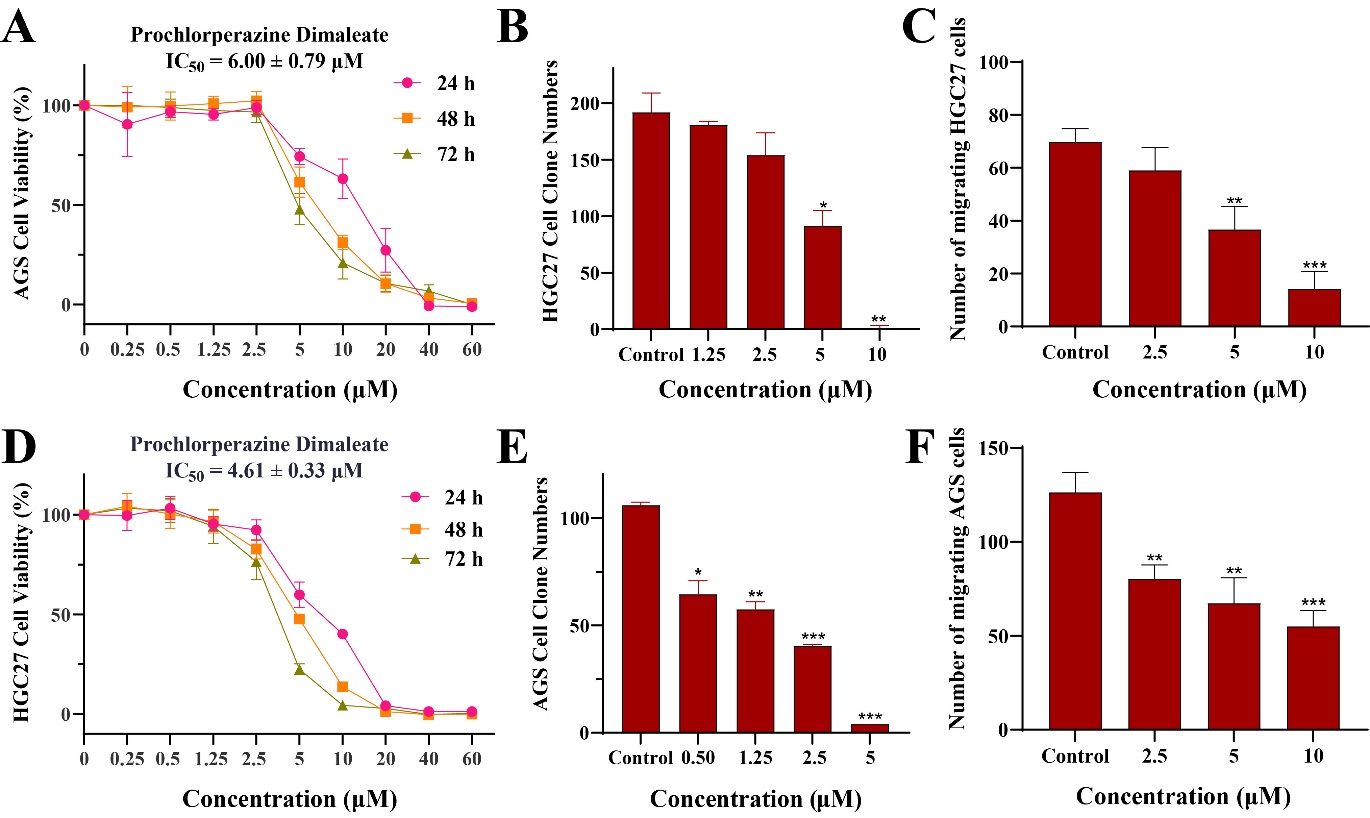


**Figure S1. PD exerted good cytotoxicity in different cancer cells and inhibited the formation of colony and migration of AGS and HGC27 cells. (A)** Cytotoxic effects of PD against gastric cancer AGS cells for 24 h, 48 h, 72 h. **(B)** Quantification of colony formation area of HGC27 cells is expressed compared with vehicle control after treatment for 8-10 days. **(C)** The number of migrating HGC27 cells were quantified after the treatment of PD in different concentrations. **(D)** Cytotoxic effects of PD against gastric cancer HGC27 cells for 24 h, 48 h, 72 h. **(E)** Quantification of colony formation area of AGS cells is expressed compared with vehicle control after treatment for 8-10 days. **(F)** The number of migrating AGS cells were quantified after the treatment of PD in different concentrations. Values are shown as the means ± standard, n = 3. **p* < 0.05, ***p* <0.01, ****p* < 0.001 compared to negative control group.


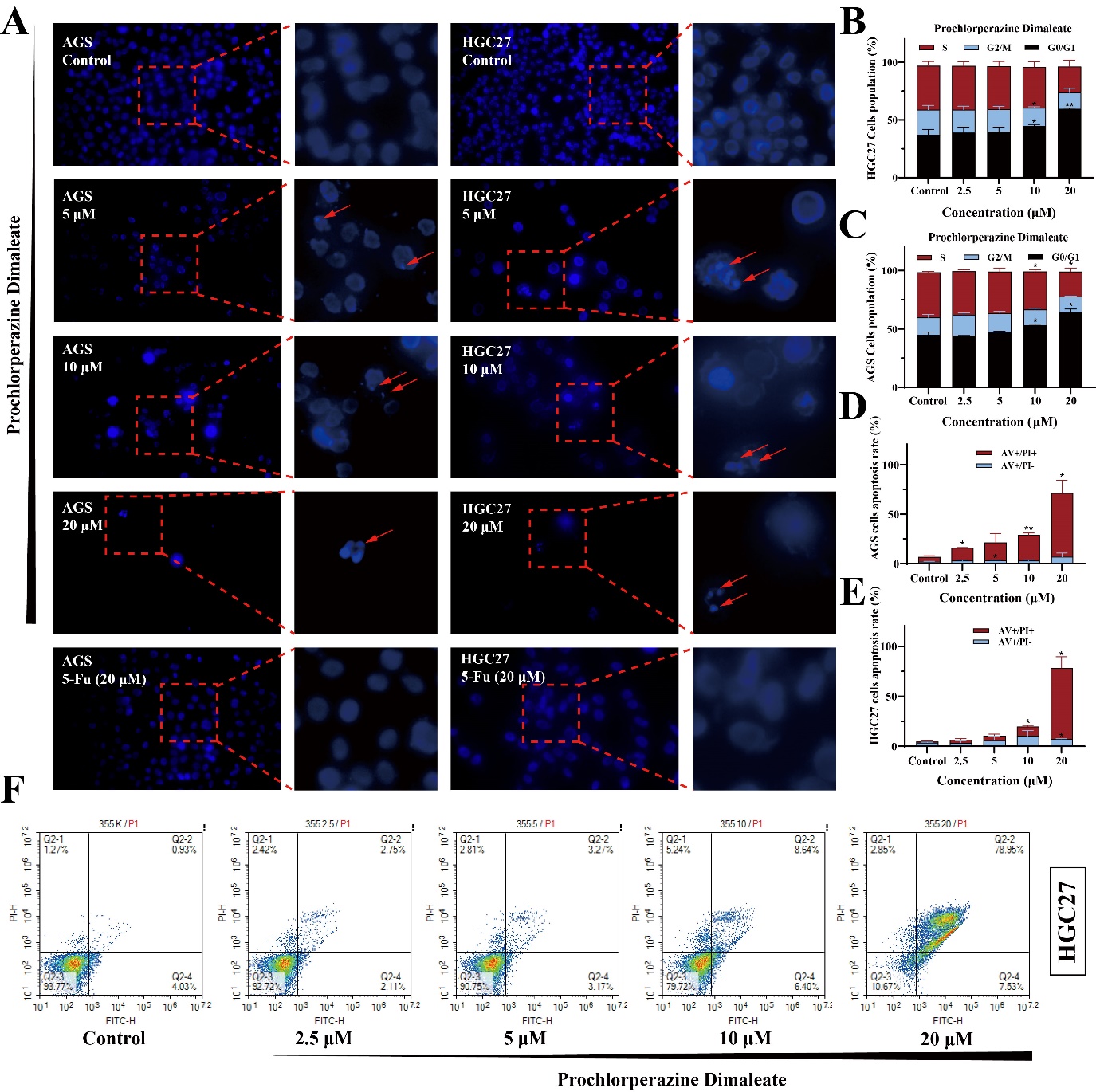
 **Figure S2. PD arrested cell cycle at G0/G1 phase and induced cells apoptosis of gastric cancer AGS and HGC27 cells. (A)** PD induced nuclear shrinkage of AGS and HGC27 cells and produced bright blue spots after the treatment for 48 h. **(B) and (C)**The distribution of the cell cycle of HGC27 cells and AGS cells was examined after the treatment of PD for 24 h in different concentrations. **(D) and (E)** The ratio of apoptosis in AGS and HGC27 cells after the treatment with different concentration of PD for 48 h. **(F)** PD induced apoptosis of HGC27 cells after the treatment for 48 h. Values are shown as the means ± standard, n = 3. **p* < 0.05, ***p* <0.01, ****p* < 0.001 compared to negative control group.


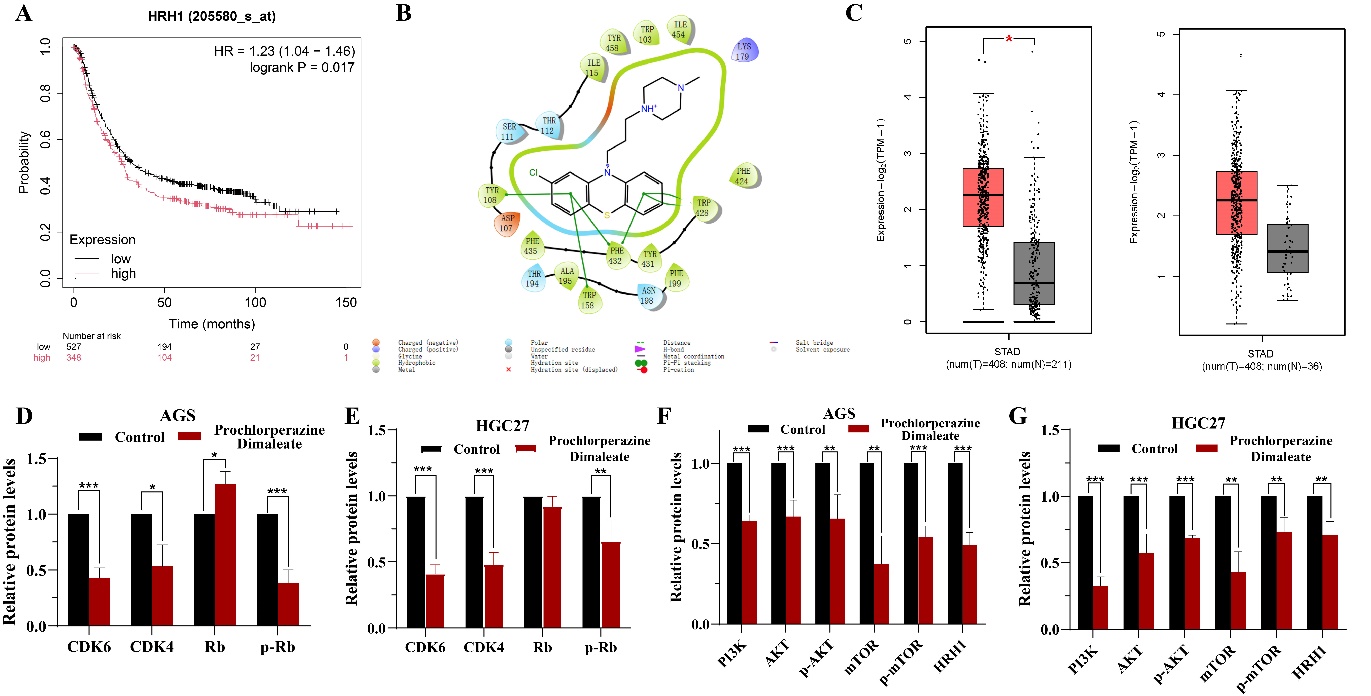
 **Figure S3. Molecular docking of PD and HRH1 protein and bioinformatics analysis of HRH1 mRNA in gastric cancer patients and normal tissues. (A)** Patients with high expression of the HRH1 protein have a poorer prognosis. **(B)** 2D diagram of HRH1 interacting with small molecule ligand PD. **(C)** Expression of HRH1 mRNA in gastric cancer patients and normal tissues. **(D)** **and (E)** PD inhibited the expression of proteins associated with cells cycle in AGS and HGC27 cells for 48 h. **(F) and** **(G)** PD inhibited the expression of proteins associated with PI3K/AKT/mTOR cell signaling pathway in AGS and HGC27 cells for 48 h. Values are shown as the means ± standard, n = 3. **p* < 0.05, ***p* <0.01, ****p* < 0.001 compared to negative control group.


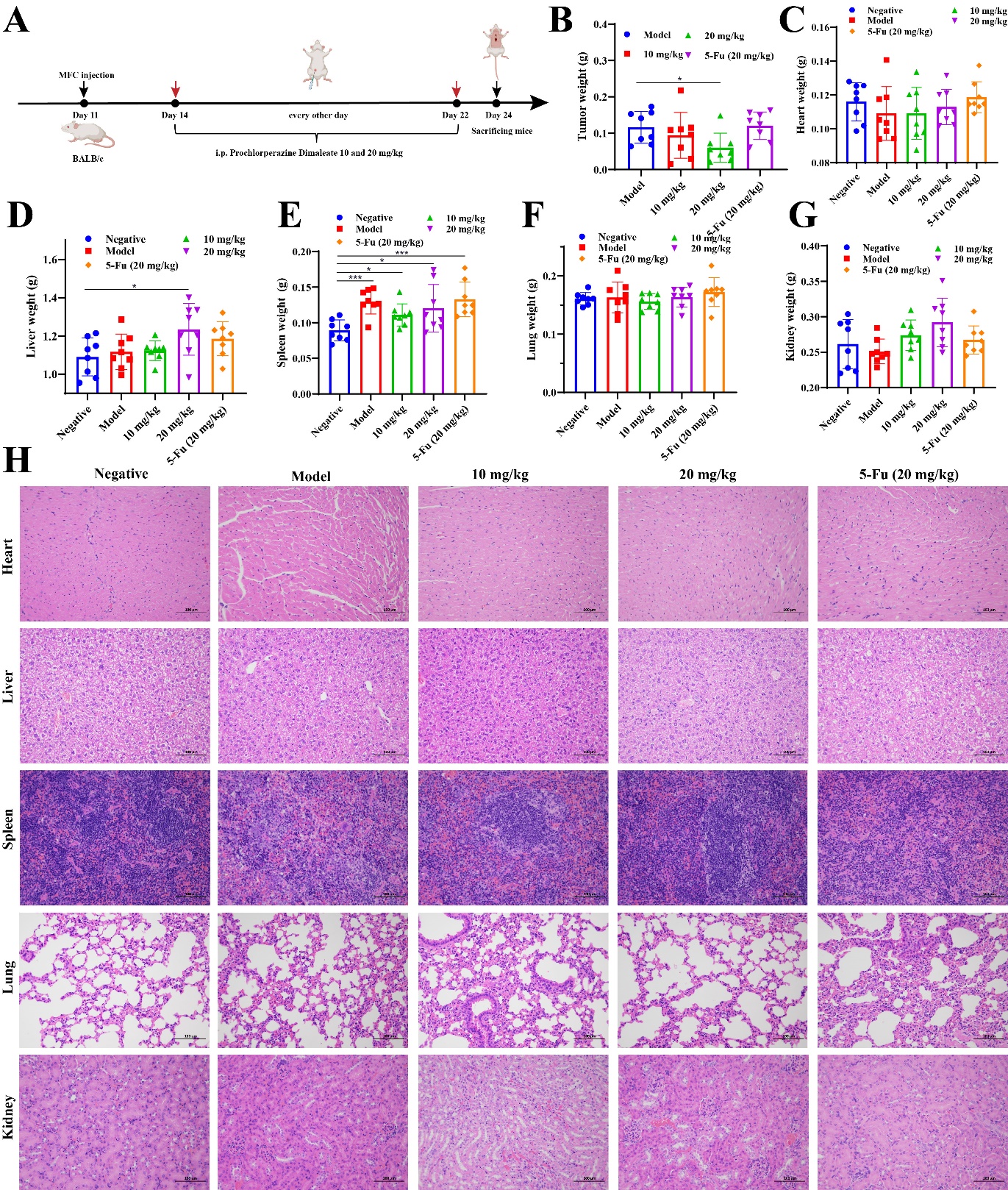


**Figure S4. PD exerted good anti-gastric cancer effect *in vivo* and low toxicity to the major organs of BALB/c mice. (A)** The timeline of the *in vivo* anti-gastric cancer experiment with PD. **(B)** The tumor weight of BALB/c mice in different group after the treatment of PD or 5-Fu. **(C-G)** The weight of heart, liver, spleen, lung, kidney of BALB/c mice in different groups after the treatment with PD or 5-Fu. **(H)** The H&E staining analysis of main organs (heart, liver, spleen, lung, kidney) in BALB/c mice after treatment with PD or 5-Fu.
